# Preschoolers’ parent and teacher/director perceptions of returning to early childcare education during the COVID-19 pandemic

**DOI:** 10.1186/s12889-022-14409-w

**Published:** 2022-12-05

**Authors:** Meg Bruening, Camila Nadalet, Nathan Ashok, Bin C. Suh, Rebecca E. Lee

**Affiliations:** 1 Department of Nutritional Sciences, College of Health and Human Development, The Pennsylvania State University, Chandlee Building, University Park, PA 16803 USA; 2 Center for Health Promotion and Disease Prevention, Edson College of Nursing and Health Innovation, Arizona State University, 500 N 3rd Street, Phoenix, AZ 85004 USA; 3 Bureau of Assessment and Evaluation, Arizona Department of Health Services, 150 N 18Th Ave, Phoenix, AZ 85007 USA

**Keywords:** Preschool, COVID-19, Perceptions

## Abstract

**Background:**

Early Care and Education (ECE) sites are critical hubs for social, emotional, and physical learning development of preschool children (ages 3–5). The COVID-19 pandemic has impacted ECE enrollment and participation; until June 2022, preschool children in the US were ineligible for COVID-19 vaccines. It is critical to identify perceptions of teachers/directors and parents to enhance safe return-to-school efforts.

**Methods:**

Focus groups (*n* = 7; 22 participants) were conducted with ECE teachers/directors throughout Arizona to examine perceptions of COVID-19 testing for families and staff at ECE sites, and current and possible COVID-19 mitigation strategies during Summer 2021. Preschool parents from underserved families in Phoenix (*n* = 41) completed a brief survey on their perceptions of benefits of ECE for themselves and their children, thoughts on COVID-19 mitigation strategies, and timing for safe return to school during Spring 2021. Focus groups were transcribed and analyzed for themes using constant comparison.

**Results:**

There were 4 focus group themes: 1) perceptions of saliva-based COVID-19 testing, 2) logistical strategies for COVID-19 testing at ECE sites; 3) successes and challenges with current COVID-19 mitigation strategies; 4) ideas to support improved COVID-19 mitigation, including outdoor gardening. Parents rated peace of mind about the child’s education as the most important benefit for themselves of in-person ECE (74.6%), and social development for children as the most important benefit for their children (54.4%). Over 40% of parents reported it would not be safe to send children back until 2022.

**Conclusions:**

COVID-19 continues to impact attendance at ECE sites, despite parents reporting key benefits to attending ECE sites. Teachers/directors supported COVID-19 mitigation strategies including saliva-based testing and gardening education to improve safe return to schools.

## Introduction

Preschool-aged children, especially those in underserved communities, have experienced increased deficits in learning opportunities due to COVID-19 [1–3]. In addition to literacy, numeracy and other academic skills, early childhood is a period of intense motor development and nutritional needs, and many families from underserved populations rely on early care and education (ECE, i.e., preschool) to help meet these developmental needs [4, 5]. The pandemic caused schools and ECE sites throughout the US to shift to emergency remote learning, which typically reduces learning opportunities and engagement [6]. Most children have spent more time online [7]. With ECE site closures, children’s physical activity has decreased, while screen time has increased, for both education and recreation purposes [7–9], worsening already too low amounts of physical activity, potentially impeding motor development [8, 10]. Adults have reported more unhealthy eating behaviors during the pandemic [11]. Avoiding dine in restaurants has sent many to drive through fast food take out windows, and higher fast food consumption has been associated with poverty and obesity [12], contributing to poor nutrition for young developing brains and bodies. As the pandemic has persisted, more information is needed to understand how the pandemic has impacted lifestyle behaviors, particularly among those with young children who have attended ECE sites previously.

In some places, including Arizona, many ECE sites serving low-income and underserved students were closed through 2021, further limiting young children’s access to education. As these sites have reopened, children and staff (e.g., teachers) at ECE sites are increasingly affected by COVID-19 infection. At the onset of the pandemic, it was unclear how young children could contract or spread COVID-19. However, recent data from ECE settings shows that children can *not only* contract SARS-CoV-2 in ECE environments, *but also* spread it to others, even when they are asymptomatic [13–16]. ECE staff also pose high risk of virus transmission within the ECE environment [14], possibly because adults have been found to carry higher viral loads than children, leading to an increased incidence of transmission [14]. Research is needed to better understand how to best mitigate COVID-19 in ECE environments, where the COVID-19 mitigation strategies are different due to the developmental stage of the children (e.g., possible difficulty wearing face masks, understanding of infectious disease).

The Centers for Disease Control and Prevention (CDC) promotes a layered prevention approach against COVID-19 for ECE sites: vaccination for eligible individuals, masking, and social distancing [17–20], as well as, use of adequate indoor ventilation and outdoor learning. Social distancing is presently known as an effective COVID-19 prevention measure [21], and public masking in Arizona schools showed a significant decrease in transmission of COVID-19 [19]. Until Fall 2022, positive cases were being reported frequently in ECE sites across the US [1, 22, 23]. Thus, these established mitigation strategies may be more effective when combined with testing school staff and children for COVID-19 infection periodically, perhaps as part of back-to-school readiness, in order to greatly reduce transmission risk [24, 25]. It is unclear how COVID-19 mitigation strategies, including onsite testing, would be perceived by those working in ECE sites and the parents of children attending the sites. Understanding perceptions as they relate to testing would help to better understand risk for COVID-19 for children at ECE sites.

Persistent disparities in infection and subsequent morbidity and mortality have affected underserved populations (low-income and communities of color [26, 27]), especially in Arizona [28]. Specifically, people in Arizona who are American Indian, Hispanic, or Black have higher rates of COVID-19 cases, hospitalization, and deaths compared to people who are White [28]. Despite these documented disparities, there has been little focus on young children. This study aimed to identify and understand the best practices of ECE sites that were operating and how to best support these practices to help keep children from underserved populations safely in school as the pandemic continues to help close education deficits. The aim of the present study was to explore ECE teacher/director perceptions COVID-19 mitigation strategies and a secondary aim was to understand parents’ perceptions of ECE sites during the pandemic as well as self-reported preschool children and their families’ nutrition and physical activity behaviors and practices during the pandemic.

## Materials and methods

We conducted a concurrent mixed methods study to explore parent and teacher/director perceptions of COVID-19 mitigation strategies and the impact on ECE children and sites. In the Spring of 2021, we administered a survey to parents of ECE children who had previously participated in our NIH-funded gardening intervention, Sustainability via Active Garden Education (SAGE) [29], at ECE sites serving low-income families in the Phoenix metropolitan (NCT03261492). A total of 363 parents were called, of these, 29 (9.0%) refused, 22 (6%) were no longer in service, 249 (69%) did not respond, and (16%) 59 were reached, with 55 that had complete information for analysis.

As sites began to reopen, in the summer of 2021, we conducted focus groups over Zoom with teachers and directors at ECE sites serving underserved populations throughout Arizona, in preparation for an upcoming trial, Back to Early Care and Education Safely with Sustainability via Active Garden Education; BE SAGE; (NCT05178290). A purposive sampling approach was used in which teachers/directors at ECE sites were recruited for the focus groups, using listserv messages, personal invitations, and word of mouth. Inclusion criteria included working at an ECE site in Arizona prior to the pandemic and participating the Child Adult Food Program. As such, all types of centers (e.g., Head Start Programs, home-operated centers) were included in the study. In order to get a broad understanding of experiences, participants came from ECE sites that had already reopened as well as sites that were still closed. To minimize spread of COVID and to increase reach to potential participants, focus groups were conducted and recorded on Zoom. All participants provided written informed consent. Parents were thanked for their time. Teachers/directors received a $50 gift card for their time. All protocols were approved by the Arizona State University Institutional Review Board (protocol ID: 00,003,761).

### Measures

#### Teacher/director focus group

A total of seven focus groups were conducted and recorded until saturation in June and July 2021 using Zoom. Participants had the choice to turn their video on or off, and only audio recordings were saved. Audio recordings were automatically transcribed by Zoom, and checked for accuracy by trained research assistants. A semi-structured focus group guide assessed was used in each focus group assess ECE teacher/directors (*n* = 22) perceptions of saliva COVID-19 testing onsite for children, their families, and teachers and staff. Teachers/directors also reported on their thoughts about current success and challenges with COVID-19 mitigation strategies, perceptions of vaccine acceptance among teachers at their site, and possible ideas for future COVID-19 mitigation approaches including outdoor gardening education for children. Physical activity and other health behaviors were not examined in the focus groups. All participants were women. Additional demographic data were not collected on the teachers/directors.

#### Survey measures

In March 2021, a convenience sample parents were contacted by phone and asked to complete a brief survey via interview developed for this study to assess their comfort with sending their children back to in-person ECE. The questions examined perceptions about benefits of returning to ECE sites, mitigation strategies, and a safe timeframe to return to in-person ECE (Table 1). When parents responded to these questions, they were asked to rank their top 3 selections. Questions were asked to assess changes in work, dietary habits, physical activity, and screen time for members of the household (Table 1). Parental demographic data (sex, Hispanic ethnicity, marital status, education, and age) were also collected.

**Table 1:** Survey questions asked of preschoolers’ parents on their experiences and perceptions about COVID

Construct	Question	Response options
Perceptions about early childcare education	What benefits do you, as a parent, experience when your kids are in class-room based school? Please select all that apply	I worry less about their safety It gives me time to work we all get along better at home Having children at school eases a financial burden I worry less about how Im doing as a parent or my parenting skills I have more peace of mind about their learning I have more personal space
	What benefits does your child/children experience when they are in class-room based school? Please select all that apply	Kids get to see their friends Kids get more physical activity at school Kids sleep better at night when they go to school during the day Kids learn more at class room based school Kids are happier at home when they are able to go to school Children have more structure to their days when they are in school Children gain more social development skills when they are in school Other
Perceptions about COVID mitigation strategies	We want to ask you about strategies for getting back to school. Tell me, which strategies do you think would be important for getting kids back to school safely? Please select all that apply	Disinfecting classrooms daily Screening teachers for COVID 19 weekly Shorter school hours, like only morning school to avoid lunch hour and recess Vaccinating teachers Making sure kids are wearing masks or face covering Keeping kids and teachers a safe distance apart Regularly screening families of kids at your school Testing kids and families who get sick for contact Weekly school to parent communication Requiring parents and/or other adult family member Vaccinating children for COVID 19 Requiring children to get their flu shot School ventilation system upgrades Nothing would make me feel safe about returning my child back to school
	When do you imagine that it will be safe to send kids back to classroom-based school again, like it was before the pandemic? (Month/Year)	Open ended question
Work changes due to COVID	As a result of the COVID-19, how has your work (for wages) changed?	I have a lot more work I have a little more work I have about the same amount of work I have less work I have no work
Physical activity, screen time, and sedentary behavior changes due to COVID	How has the COVID-19 pandemic changed your level of physical activity? Physical activity might be things like going to the gym, walking or riding bicycles, taking an exercise, yoga or activity class	I am doing a lot more physical activity I am doing a little more physical activity I am doing about the same amount of physical activity as usual I am doing a little less physical activity I have stopped doing most physical activity
	How much would you agree with the statement: I have started using an app on my phone to monitor my physical activity or walking?	Strongly agree Agree Neutral Disagree Strongly disagree
	How much would you agree with the statement, I have started using virtual exercise, yoga, or other activity classes on my phone, computer or TV	Strongly agree Agree Neutral Disagree Strongly disagree
	How has the COVID-19 pandemic changed your child’s level of physical activity?	My child is doing a lot more physical activity My child is doing a little more physical activity My child is doing about the same amount of physical activity My child is doing a little less physical activity My child has stopped doing most physical activity
	How much would you agree or disagree with the statement: my child or children spend more time on schoolwork than a cell phone, tablet?	Strongly agree Agree Neutral Disagree Strongly disagree
	How much would you agree or disagree with the statement: my child or children spend more time watching television shows or movies since the pandemic?	Strongly agree Agree Neutral Disagree Strongly disagree
	How much would you agree with the statement: my child or children spend more time playing games on a cell phone, tablet, or computer?	Strongly agree Agree Neutral Disagree Strongly disagree
Dietary changes due to COVID	How much would you agree with the statement: my family and my eating habits have improved, like eating more fruit and vegetables and less fried or takeout food?	Strongly agree Agree Neutral Disagree Strongly disagree
	How much would you agree with the statement: My family and I have been eating take-out food more than usual?	Strongly agree Agree Neutral Disagree Strongly disagree
Weight changes due to COVID	How much would you agree with the statement: my family and I have started trying to lose weight	Strongly agree Agree Neutral Disagree Strongly disagree

#### Focus group analyses

Research assistants transcribed audio recordings to text. A code book using a constant comparison approach was created for the themes and the transcriptions were analyzed in which coders identified key themes and updated the codebook throughout the coding process so that there was consistency between the coders. All analyses were conducted using NVivo Qualitative Analysis Software (version 12, March 2020 release) by CN and MB. Any discrepancies in coding were discussed, and a consensus was reached.

#### Survey analyses

Descriptive (percent) statistics were used to examine parent responses to the survey.

## Results

### Teacher/director focus groups results

Seven focus groups were conducted with a total of 22 participants, with an average of 3 participants per group. There were a total of 4 themes in the focus groups: 1) perceptions of saliva-based COVID-19 testing, 2) ideas about logistics for COVID-19 saliva testing for preschoolers, their parents, teachers, and staff; 3) successes and challenges with COVID-19 mitigation strategies; 4) ideas to support improved COVID-19 mitigation strategies. At the time of the focus groups (summer 2021), it is important to note that the teachers/directors reported that 25–80% of their ECE colleagues were already vaccinated.

### Perceptions of saliva-based COVID-19 testing

In general, teachers/directors were excited about saliva-based testing for all groups including the children, families, and ECE teachers. The teachers/directors appreciated that saliva testing would be free and non-invasive, especially given experiences with the nasal swabs: “*They're child[ren], maybe they're going to be crying with the nose test. I think when they are going to hear the possibility of testing with a saliva they are going to be yes, do this to my child*.” And, “*I do like the fact that it would be a spit test versus the nasal swab, so I think a lot more parents, even myself as a parent I would feel more comfortable*.” Several respondents indicated saliva testing would be fun, such as this sentiment: “*I think the kids would be interested in it. Before COVID we used to brush our teeth at least once a day, and they got to be very good with spitting in the little spit cups and that kind of thing it was it was it was fun for them, so I don't think they would be bothered by it.”* Moreover, paricipants appreciated the importance of the information gained from the tests. As one teacher said, “*The kids that come into our care knowing, you know that we're all going to be safe and that…I think it would be nice to know if anyone, if anyone is feeling unwell [they are not sick with COVID], so I like the idea*.” Another discussed the importance of parents knowing: “*And it’s like the parents, they need to be informed. [So] they can be encouraged to test their children*.” And, “…*our district has a screening process, and then they have to be sent home, and then they either have to quarantine for the X amount of days, or they have to come back with the negative test. And this would eliminate that, it would also be really convenient for the families to just be like ‘Okay um, I know my child doesn't have COVID*.’”.

Teachers/directors worried about confidentiality of testing, especially as it relates to positive cases, and wondered whether parents would think it’s helpful. Some worried that having genetic material collected may be a barrier: *“I think that, like their DNA being out there, I think it would give them some kind of fear about it.”* There was also concern of the information being informally shared: “*Very confidential, I mean because some of our parents like to be in other people's business, so I think it should be kept confidential and be very respectful from- of it*.” The lack of concern as it related to COVID exposure to preschool aged children was raised: “*Well, I kind of think it's the way people don't want to get the vaccine… some of them just have very, very strong beliefs about that. Some of our parents are very young and they don't think it's going to happen to them, so uh, I think that is-is where it is. They're not just, they're just really not sure about what it is, as far as is it going to affect their children later*.”

### Dealing with positive cases (saliva-based COVID-19 testing sub-theme)

All of the particpants reported that their ECE sites had policies for positive cases, which were usually guided by state and federal guidelines. For example, one teacher said, “*Well, I had a child test positive, so you basically have to just go ahead and shut down, like do the 10 day and have everyone test. Like, just go just go based on the CDC guidelines of what happens when someone's positive.*”

### Logistical considerations for COVID-19 saliva testing

Participants shared that, at least in Arizona, parents were not allowed in ECE buildings and/or classrooms to minimize possible COVID-19 exposure to children. As a result, most of the testing would need to take place outside, during drop-off and/or pick-up, depending on the site: “*I think bringing it to the school and doing it. Like, if you're doing it at drop off time you're going to have some things set up right outside, parents can go there before they pick up their child, do what they need to do, or, bring their child whatever-however you guys are going to do it, but I think having it at you know, because we have different sites so having it at each site, I think that you're going to get most parents that way*.” “*I feel like at drop-off would be the best time too because we have we have a little office where we drop off the kids and, there's empty office space where testing could be done. So it could be a child at time, and um, there's I think there's even more than one office space open so I’m just thinking about that at drop off would be the best time for our site*.” Some discussed being sure to include the onsite health professionals to help organize the testing: “*For my site, they have nurse and if you get contact with her, they will provide us a-a room for you can be testing child by child*.”

### Successes and challenges with COVID-19 mitigation strategies

ECE teachers/directors indicated quite a bit of success with the current mitigation approaches, especially hand washing and mask wearing: “*Hand washing, we do a lot of a hand washing we are forever washing our hands. And they're used to it. They're used to the routine we come in, wash our hands, go outside, play, come in, wash our hands before we eat. You know they sneeze or cough, they're washing their hands, so they're really into that*.” Another teacher: “*For our class, um, the most successful has been wearing the mask with the children. At first initially I thought that they were going to have a hard time, but they have been so fantastic at wearing their masks.”*

Others discussed temperature checks, sanitizing, and social distancing, as new challenges for their sites. One respondent pointed out that, “*For me is the whole, uh, temperature checks it's still confusing because a lot of people that have had COVID, don't get a fever. So it's hard to determine if somebody has COVID.”* The teachers acknowledged an increase workload related to sanitizing efforts: *“I think it's added more work to our job as far as not that we didn't keep the place clean, but you are more- you're cleaning on top of cleaning, if-if you will… we had Plexiglass dividers on a lot of our tables; those are difficult to you know, to keep clean.”* Sometimes, the workload was balanced with lower classroom student-to-teacher ratios: *“We didn't do in depth cleaning like we do things now. So I mean…it's definitely gotten a lot easier now, I would say just because we're used to it…And we have less kids, so because of the ratios of being down, the classroom is honestly less worn out and we’re able to sanitize a lot more frequently as well. So with smaller ratios it's been a lot easier to upkeep a healthier environment as well….”*

Subthemes in COVID-19 mitigation successes and challenges included ECE site teachers/directors speaking about the effects that mitigation strategies have had on their classrooms in terms of the challenges related to caring for young children and students continuing to get sick. They also discussed future challenges related to those outcomes such as when they must return to larger class sizes again.

### Struggling with strict illness policies at ECE sites

The participants described the implications of requirements for sending children home if they had any cold or flu symptoms. One poignant quotation about the struggle from both the teacher and the parent perspective was: *“….on our end it's been a little difficult because we hate like being those bad people you know, having to send your child home… we don't want our parents to lose their job at the end of the day, because we do have to hear those stories where parents is bugging us on the phone saying, ‘I have no one, you know.’ ‘I'm going to lose my job if I pick up my son today, how can you do this to me?*’ *And from my end, you know and I’m like I’m a parent too, I definitely understand their point of view but it's like if I continue to have their child here that can’t contain their cough…can't enforce a mask policy on them either. It’s just very hard as well because you don't want to risk other children and other parents to be in the same situation if you don't send this child home.*”

### The impact of COVID-19 mitigation strategies on learning and social development

Several participants indicated concern regarding the mitigation strategies on child development. For example, one indicated: “*I just feel that the kids are missing out on some things, as I said before, we don't brush our teeth this year. I think that was a good thing for our Head Start kids but, we didn't feel it was safe to do that kind of thing you know, considering what was, what was going on*.”

### Ideas to support improved COVID-19 mitigation strategies

Although the ongoing mitigation strategies seemed to be routine for many of the teachers, there were some ideas to help teachers be more effective in managing the extra workload. For example, some teachers recommended creating checklists to help ensure that all strategies were successfully implemented: “*Sometimes, something happens out of our day that's not normally part of our routine and then we forget where we were at and it could throw us off. And just having like a checklist or something that's there to go off of, you can be like ‘Oh, you know, this is where I was at, let me continue’, and you don't have to feel like your whole day’s messed up and you feel like, a chicken with your head cut off*.”

### Outdoor gardening as a mitigation strategy

A subtheme that emerged as a novel mitigation strategy was to use outdoor gardening education. Teachers/directors were enthusiastic about gardening education. Teachers liked that gardens would expose children to fruit and vegetables, help get kids outside, and help educate parents: “*It’s a great learning experience for kids to-to see plants grow and then to harvest them*.” Another teacher provided, “*I think that if we had all the materials to do it and we had like a guideline of this week we're going to do this, and this. It makes it a lot easier on teachers, I think teachers would be more open to doing it, and I think the kids will definitely get into it as well*.”

### Parent survey results

In total, 55 recent SAGE parents (*M* = 34.5 ± 8.23 years) responded to the survey. Most parents were women, Hispanic, and married or living with a partner (Table 2). The top benefit parents reported for having classroom-based school for their children included having more peace of mind about their learning (74.6%), gives the parents time to work (42.4%), having children at school eases financial burdens (32.2%), and allows parents more personal space (32.2%; Table 3). Parents rated social development as the most important benefit of in-person ECE for children (54.4%). However, many parents have been reluctant to send children back to preschools–42% of SAGE parents surveyed believed it would not be safe to send children back until sometime in 2022.

**Table 2:** Demographic characteristics of parents who completed surveys

**Variable**	**Total** *N* = 55 N (%)
Parent sex	
Male	5 (9.3)
Female	49 (90.7)
Hispanic	39 (73.6)
Marital status	
Married	17 (37.0)
Living with a partner	11 (23.9)
Separated, divorced, widowed	8 (17.4)
Single (never married)	10 (21.7)
High school/GED or higher	37 (69.8)
Age (year), M (SD)	34.5 (8.23)

**Table 3:** Preschool parent responses to the impact of COVID-19 on perceptions of early childhood education centers

**Variable and response options**	% (n)
**Top 3 ranked benefits for parents for classroom-based school**	
I have more peace of mind about their learning	74.6 (44)
It gives me time to work	42.4 (25)
Having children at school eases a financial burden	32.2 (19)
I have more personal space	32.2 (19)
We all get along better at home	30.5 (18)
I worry less about their safety	27.1 (16)
I worry less about how I am doing as a parent or my parenting skills	18.6 (11)
**Top 3 ranked benefits for kids for classroom-based school**	
Children have more structure to their days when they are in school	54.2 (32)
Children gain more social development skills when they are in school	54.2 (32)
Kids get to see their friends	47.5 (28)
Kids learn more at classroom-based school	47.5 (28)
Kids get more physical activity at school	25.4 (15)
Kids sleep better at night when they go to school during the day	20.3 (12)
Kids are happier at home when they get to go to school	20.3 (12)
**Top 3 ranked strategies for getting kids back to school safely**	
Disinfecting classrooms daily	61.0 (36)
Making sure kids are wearing masks or face coverings	28.8 (17)
Keeping kids and teachers a safe distance apart	27.1 (16)
Vaccinating teachers	23.7 (14)
Weekly school to parent communication about what I…	20.3 (12)
Vaccinating children for COVID-19	20.3 (12)
School ventilation system upgrades	18.6 (11)
Screening teachers for COVID-19 weekly	16.9 (10)
Requiring parents and/or other adult family member	13.6 (8)
Shorter school hours, like only morning school to avoid lunch hour exposure and sites	11.9 (7)
Regularly screening families of kids at your school	8.5 (5)
Testing kids and families who get sick for contact	8.5 (5)
Requiring children to get their flu shot	5.1 (3)

In total, 45% of parents reported working less as a result of the pandemic (Table 4). Physical activity decreased for many parents, with 48.2% reporting a decrease, and 25% reporting no change in physical activity. Parents (56.3%) reported using apps to monitor physical activity and 38.9% used virtual physical activity classes. Half of the parents reported that children were doing less physical activity as well as a result of the pandemic and almost 30% reported no change in their child’s physical activity. Parents reported more screen time as a result of COVID-19 with 44.4% agreeing that there were higher amounts of cell phone/tablet use by their children, 62.2% agreeing that their children were watching more shows/movies, and 52.9% reporting more time playing electronic games. For these families, 53.7% reported improved eating habits, and 33% reported having more takeout food.

**Table 4:** Preschool parent responses to the impact of COVID-19 on changes in work hours, physical activity, screen time, and dietary behaviors

Variable and response options	% (n)
**Change in work hours as a result of COVID**	
I have a lot more work	12.5 (7)
I have a little more work	12.5 (7)
I have about the same amount of work	30.4 (17)
I have less work	19.6 (11)
I have no work	25 (14)
**Changes in parent physical activity due to COVID-19**	
I am doing a lot more physical activity	8.9 (5)
I am doing a little more physical activity	17.9 (10)
I am doing about the same amount of physical activity as usual	25 (14)
I am doing a little less physical activity	35.7 (20)
I have stopped doing most physical activity	12.5 (7)_
**Changes in child physical activity due to COVID-19**	
My child is doing a lot more physical activity	9.3 (5)
My child is doing a little more physical activity	11.1 (6)
My child is doing about the same amount of physical activity	29.6 (16)
My child is doing a little less physical activity	35.2 (19)
My child has stopped doing most physical activity	14.8 (8)
**Parent started using phone apps for monitoring physical activity**	
Strongly agree	14.5 (8)
Agree	41.8 (23)
Neutral	10.9 (6)
Disagree	18.2 (10)
Strongly disagree	14.5 (8)
**Parent started using virtual physical activity classes**	
Strongly agree	14.8 (8)
Agree	24.1 (13)
Neutral	16.7 (9)
Disagree	33.3 (18)
Strongly disagree	11.1 (6)
**Child/children are spending more time on schoolwork then on screens**	
Strongly agree	18.5 (10)
Agree	25.9 (14)
Neutral	27.8 (15)
Disagree	20.4 (11)
Strongly disagree	7.4 (4)
**Child/children spending more time watching TV or movies**	
Strongly agree	24.5 (13)
Agree	37.7 (20)
Neutral	22.6 (12)
Disagree	13.2 (7)
Strongly disagree	1.9 (1)
**Child/children spends more time on cell phone, tablet, or computer**	
Strongly agree	20.8 (11)
Agree	32.1 (17)
Neutral	20.8 (11)
Disagree	20.8 (11)
Strongly disagree	5.7 (3)
**Family’s eating habits have improved**	
Strongly agree	9.3 (5)
Agree	44.4 (24)
Neutral	29.6 (16)
Disagree	13 (7)
Strongly disagree	3.7 (2)
**Family has been eating more takeout food**	
Strongly agree	3.7 (2)
Agree	29.6 (16)
Neutral	20.4 (11)
Disagree	35.2 (19)
Strongly disagree	11.1 (6)
**Family has started to lose weight**	
Strongly agree	22.2 (12)
Agree	42.6 (23)
Neutral	13 (7)
Disagree	13 (7)
Strongly disagree	9.3 (5)

## Discussion

The dual purpose of this mixed-methods study was to explore perceptions of how to return young children safely to ECE and understand how family health behaviors may have changed during the COVID-19 pandemic. Given that COVID-19 vaccines only became available for preschool children in June 2022, and COVID-19 variants are impacting young children at an increasing rate [1, 22, 23], this study offers a better understanding of testing and mitigation strategies to encourage enrollment and ongoing attendance in ECE sites is needed. Teachers reported high support for non-invasive saliva COVID-19 testing and thought ECE sites would be ideal for implementation of the testing. For those who had taught in the 2020–2021 school year, teachers reported schools and children adapting to COVID-19 mitigation strategies relatively easily; although, some had concerns about the developmental implications for the children and the extra time it took to implement the strategies. Teachers enthusiastically supported outdoor gardening as a COVID-19 mitigation strategy for preschool children. Parents reported high value in their children attending ECE sites, but strong hesitation in returning their children to ECE sites before 2022.

Mitigation of COVID-19 is ever-evolving as new strains of the virus influence the infectiousness of the disease, and as vaccines roll out to younger populations. Beyond vaccinations, evidence consistently shows that good ventilation, social distancing, proper mask wearing, regular handwashing, and staying home when sick are helpful in decreasing risk of exposure to COVID-19 for all populations [24]. The teachers in this study reported that mask wearing and handwashing with young children has gone relatively well, especially when incorporated into the routine of the day. The teachers/directors also indicated that the children and their parents would enjoy outdoor gardening education and that it would be well accepted by parents, which is supported by our previous work [29, 30]. More research is needed to examine how effective gardening education is for mitigating COVID-19 among preschool children.

Currently, there are no saliva-based COVID-19 tests that are readily available for young children. Saliva tests are FDA approved for children under 5 years old, but, to our knowledge, no group in the US has implemented wide-scale testing of this population, but researchers in Germany successfully tested this approach [31]. We have begun to examine the acceptability and feasibility of implementing population-based saliva testing. The ECE teachers/directors in this study indicated enthusiasm for saliva testing, as saliva tests are relatively non-invasive compared to nasal swabs. According to our findings, consideration must be made on a site-by-site basis to accommodate site-level logistical constrains and onsite support (e.g., school nurses). Given the concerns about positive cases and parents potentially missing work due to sick children as indicated by the teachers in this study, it would be helpful if state or federal support was in place for paid leave for families missing work due to COVID-19. Care must be taken when implementing any COVID-19 testing with underserved populations (e.g., people of color, low-income families) given historical misuse of data [32, 33]. Based on the limited concerns highlighted in this study, clear, straightforward assurances of data protection and privacy laws need to be reiterated with families.

Consistent with other studies [34, 35], in the midst of COVID-19, parents in the current study reported ECE sites being a source of social, developmental growth for their children. Interestingly, just more than half of the parents (58%) reported being comfortable sending their child back to their ECE site before 2022. The same parents reported that a key benefit to having their children in ECE sites provides parents time to go to work. Nationally, multiple reports indicate that people, especially low-income women, are leaving the workforce and that the lack of safe childcare during the COVID-19 pandemic was a driver for some to stay out of the workforce [36, 37]. Roughly 45% of parents surveyed in this study reported having less work or no work at all as a result of the pandemic. This is consistent with reports that low-wage workers lost their jobs at five times the rate of middle-wage workers during the first year of the pandemic [24]. Many of these workers were parents of young children [25, 26], limiting access to ECE resources for their children and causing deficits in learning opportunities. At the same time, many ECE sites serving underserved communities in Arizona were closed for more than one year, and this closure likely impacted parents’ ability to return to the workforce. Future research is needed on how perceptions of safe ECE is linked to leaving the workforce, as well as how it relates to the health and development of children over time.

In the current study, parents reported a decrease in physical activity as a result of the pandemic for both themselves and their children, which is consistent with other COVID-related studies [7]. Parents also noted seeing an increase in the amount of television and movies their children were watching as well as the amount of time they spent playing games on various electronic devices. Long-term effects of decreased physical activity and increased exposure to screen time can be detrimental, and both are associated with overweight and obesity in children, an ongoing concern in the US and other countries [38, 39]. The contribution of COVID-19 to these factors further underscores the importance of safely returning children to school as the pandemic continues. Interestingly, the parents in the current study reported improved healthy eating and relatively low levels of fast food consumption. These results are contrary to the limited literature on eating during the pandemic [11, 12], and provides a first glance at how underserved households with young children are managing in these rapidly changing times. Despite these findings, more research is needed on how the pandemic has impacted health behaviors overtime for families and preschoolers.

Together, the survey results and focus group findings aligned and support the reality that ECE sites are critical venues to supporting underserved families with preschoolers with COVID-19 mitigation. Parents highlighted the need to continue to send their children to school; while teachers were eager to serve as a resource and model for families. Gray literature indicates that the pandemic exacerbated shortages in staffing for ECE sites [40, 41], likely affecting enrollment at sites serving low-income and underserved children. Many ECE sites serving underserved communities in Arizona were closed for more than one year. Study findings suggest that ECE site closures likely impacted physical activity and screen time, which in turn could impede motor skills development for many children. We anticipate that the closure impacted parents’ ability to return to the workforce, and study findings suggested more meals at home, perhaps reflecting preferences for staying safer at home or containing costs with home cooked meals. Future research should follow how these combined ECE and home environment and behavior changes impact the health and development of children over time. Additionally, as the pandemic progresses, more research is needed on the role of ECE in supporting changing factors related to COVID-19 mitigation including changes in masking, testing, and vaccination.

Although the sample of teachers/directors was purposeful, we did not collect demographic data, and therefore findings may not be generalizable to all teachers/directors in Arizona. In addition, the recruitment was open to all centers participating in the Child and Adult Care Food Program, but we did not assess from which type of center focus group participants represented. Anecdotally, teachers mentioned that they were representing home centers, Head Start sites, sites housed in elementary schools, and large multi-center ECE operations. The sample of parents participating in the survey was a convenience sample, and may not be representative of all parents of underserved children in Arizona. These were parents of preschool age children at the start of the pandemic, and by the time that the survey was conducted, their children may have moved to kindergarten. We did not collect data on the current age of participants’ children; thus, inferences are limited to parents whose children were in preschool at the start of the pandemic. As the pandemic has rapidly changed over time, the findings are likely limited to the experiences at the moment in the pandemic in which the data were collected.

## Conclusion

COVID-19 is a global pandemic that has had far-reaching consequences for everyone, but especially young children and their families. According to ECE teachers, saliva COVID-19 testing seems to be an acceptable approach to screen for COVID-19 at ECE sites. Outdoor gardening is a COVID-19 mitigation approach that needs to be explored further. While parents see the benefits of ECE sites, strategies are needed to help families from underserved populations transition back to ECE sites safely. Many young children from underserved populations may not be achieving sufficient physical activity to provide adequate stimulation to meet motor development milestones. More research is needed to improve safe back-to-school approaches for preschool children and families and ECE sites teachers and staff.

## Data Availability

The datasets used and/or analyzed during the current study are available from the corresponding author on reasonable request.
